# Clinical and genomic characterization of Chinese patients with functional high-risk multiple myeloma: A real-world validation study

**DOI:** 10.3389/fonc.2023.1110693

**Published:** 2023-03-10

**Authors:** Yu-tong Wang, Bin Chu, Tian-guan Zhou, Min-qiu Lu, Lei Shi, Shan Gao, Li-juan Fang, Qiu-qing Xiang, Xin- Zhao, Meng-zhen Wang, Kai Sun, Li Bao

**Affiliations:** ^1^ Department of Hematology, Beijing JiShuiTan Hospital, The Fourth Clinical Medical College of Peking University, Beijing, China; ^2^ Department of Hematology, Baise people’s Hospital, Baise, China

**Keywords:** multiple myeloma, functional high risk, NGS - next generation sequencing, real world, P53 mono-allelic inactivation

## Abstract

**Objective:**

Precise risk stratification is increasingly essential in the management of multiple myeloma (MM) as some standard-risk (SR) patients still exhibit similar poor outcomes as genetically high-risk (GHR) patients in the era of novel agents. It has recently been demonstrated that functional high-risk (FHR) patients, those with suboptimal response to first-line induction therapy or early relapse within 12 months, have identifiable molecular characteristics from the SR group in the CoMMpass dataset. However, these findings lack practical validation in the real world.

**Methods:**

MM cells purified by CD138 microbeads from newly diagnosed MM (NDMM) patients received fluorescence *in situ* hybridization and sequencing with a 92-gene Panel. Cytogenetic abnormalities defined GHR patients with t(4;14) or t(14;16) or complete loss of functional P53 or 1q21 gain and International Staging System (ISS) stage 3. SR group was patients who did not fulfill any criteria for GHR or FHR.

**Results:**

There were 145 patients with NDMM, 78 in the SR group, 56 in the GHR group, and 11 in the FHR group. In the FHR group, eight patients were suboptimal responses to induction therapy, and three relapsed within 12 months. We found that male patients, patients with extra-medullary plasmacytoma (EMD), circulating clonal plasma cells (CPC) ≥0.05%, and P53 mono-allelic inactivation were significantly higher in the FHR group compared to the SR group. After a median follow-up of 21.0 months, the median progression-free survival (PFS) and overall survival (OS) were 5.0 months, 19.1 months and 36.6 months in the FHR, GHR, and SR groups, respectively. Compared to the SR group, FHR patients had a higher frequency of mutations in *MKI67*, *ERN1*, and *EML4*. GO analysis showed that mutations in FHR were enriched for oxidative stress, chromosomal segregation, and hypoxia tolerance.

**Conclusion:**

The FHR found in the SR NDMM patient group has unique clinical features, including being male, with EMD and CPC, and genetic characteristics of mutations affecting oxidative stress, chromosome segregation, and hypoxia tolerance. In contrast to previous reports, our data suggested that patients with P53 mono-allelic inactivation should be classified in the GHR group rather than the FHR group.

## Introduction

Multiple myeloma (MM) is an incurable neoplastic disease that is characterized by high heterogeneities leading to limited benefit for high-risk patients who acquired from novel agents of proteasome inhibitors (PIs) and immunomodulatory drugs (IMiDs) followed by autologous stem cell transplantation (ASCT) (1). It is critical to recognize these high-risk patients at diagnosis to improve their clinical outcomes through adaptive therapeutic approaches. The risk stratification generally applied in clinics was the revised international staging system (R-ISS) which included high-risk genetic abnormalities and biological information (2), but nearly 5-10% of low-risk patients of RISS I relapsed within 12 months with overall survival (OS) of 3 years (2). The wild detection of next-generation sequencing (NGS) of MM evaluated gene expression and mutation signatures and published profiles of UAMS GEP70, SYK92, and APOEBEC gene mutation signature to describe high-risk NDMM patients (3–6). Other clinical characteristics such as large focal lesions with a product of the perpendicular diameters >5 cm^2^, the presence of extramedullary plasmacytomas (EMD), and circulating malignant plasma cells (CPC) were also considered high-risk factors (7–9). Above all of these genetic and clinical high-risk characteristics, they have not been included in any consensus or applied in clinical practice. The second revision of the ISS (R2-ISS) and double-hit MM were established to identify further extra high-risk patients (10, 11), while patients with no adverse cytogenetic abnormalities needed to be stratified precisely.

A recent study classified functional high-risk (FHR) patients with suboptimal response to induction therapy, of whom 40% died within three years or early progressed within 12 months with a poor OS of 20.2 months (12). As high-risk cytogenetic abnormalities were not completely excluded, these FHR patients were not actually real “functional” high-risk. Based on this study, Chng et al. refined the definition of FHR without t (4;14), t(14;16), TP53 bi-allelic inactivation and gain 1q21 based ISS III (13). They evaluated genomic sequencing data and developed a machine learning classifier of FHR patients based on the compass dataset. Nevertheless, the database data used in this study lacked clinical parameters, while genetic features partially referred to R-ISS and double-hit. The values of this definition of FHR applied in the real world remain to be validated.

In this study, we retrospectively analyzed the clinical and genetic characteristics of FHR NDMM patients defined by Chng et al. in our cohort. In addition, comparing FHR and standard risk (SR) groups may help further identify high-risk patients whom high-risk cytogenetic abnormalities cannot define.

## Methods

### Patients

From October 2019 to April 2022, 145 newly diagnosed MM (NDMM) patients who received NGS detection in our department were enrolled in this retrospective study. The procedures of this study were approved by the ethics committee of Beijing Jishuitan hospital (202104-46), which the Declaration of Helsinki performed. Written informed consent was obtained from each patient before specimen detection and clinical data collection. The diagnosis, treatment, and response evaluation of MM patients were performed according to the International Myeloma Working Group (IMWG) and NCCN guidelines (14, 15). All patients with the osteolytic bone disease received bisphosphonates or denosumab monthly for at least two years. The disease staging included the ISS, R-ISS, and R2-ISS (2, 10). The general and disease information of these patients is described in **Table 1**. The EMD was detected by PET-CT, contrast-enhanced CT, or MRI. The CPC was detected by flow cytometry and defined as positive if it was higher than 0.05%. Interphase FISH (iFISH) was performed to detect the cytogenetic abnormalities, including del(17p), 1q21+, t(11;14), t(4;14), t(14;16), and t(14;20).

**Table 1:** Baseline characteristics.

	GHR, N=56	SR, N=78	FHR, N=11	*P* value	*P* value*
Age, year	65(37-88)	64(39-83)	69(45-85)	0.635	0.378
≥65岁	30(53.6%)	37(47.4%)	7(63.6%)	0.651	0.457
Sex, male/female	25/31	36/42	10/1	**0.025**	**0.009**
ECOG score, 1/2/3/4	0/18/19/19	2/22/38/16	0/3/6/2	0.612	0.627
Types of M protein				0.453	0.827
Heavy chain, IgG/IgA	24/18	33/17	6/2		
κ/λ/no secretion	7/6/1	9/16/3	3/0/0		
ISS stage, I/II/III	7/11/39	38/27/13	4/5/2	**<0.001**	0.671
R-ISS stage, I/II/III	2/35/20	33/44/1	4/7/0	**<0.001**	0.975
R2-ISS stage, I/II/III/IV	0/3/35/18	17/29/32/0	2/1/8/0	**<0.001**	0.206
Anemia	53(94.6%)	47(60.3%)	9 (81.2%)	**<0.001**	0.227
Renal dysfunction	13(23.2%)	5(6.4%)	0	**0.007**	0.412
Hypercalcemia	13(23.2%)	2(2.6%)	1 (10.0%)	**0.010**	0.225
Extramedullary disease	16(28.5%)	20(25.6%)	7 (63.6%)	**0.080**	**0.026**
CPC ≥0.05%	35(62.5%)	17(21.8%)	8 (72.7%)	**<0.001**	**0.001**
Frontline regimens				0.268	0.179
PI	24(42.8%)	36 (46.2%)	4 (36.4%)		
PI+IMiDs	32(57.1%)	42 (53.8%)	7 (63.6%)		
ASCT	15(26.8%)	23 (29.5%)	3 (27.3%)	0.769	0.897
Best response^#^				<0.001	<0.001
CR/VGPR	4/23	12/30	0/2		
PR	16	29	2		
MR/SD	2/4	0/0	2/5		

*The P value represented the comparation between SR and FHR groups.

SR, standard risk; GHR, genomic high risk; FHR, functional high risk; ISS, international staging system; R-ISS, revised ISS; CPC, circulation plasma cells; PI, proteasome inhibitor medicine including bortezomib and ixazomib; IMiDs, immunomodulator agents.

^#^Seven patients in GHR group failed to evaluate treatment response due to early death (n=5) and loss to follow-up (n=2). Seven patients in SR group failed to evaluate treatment response due to early death (n=3) and loss to follow-up (n=4). The bold values was presented for P > 0.05.

Up to January 20, 2023, the median follow-up was 21.0 months (95% CI: 19.1-22.8 months). Progression-free survival (PFS) was calculated from diagnosis to progression disease (PD) or death for any reason. Refractory patients were defined as those who had received a response less than partial response (PR) to induction therapy. All patients were divided into three groups according to the previous report: patients with t(4;14) or t(14;16) or complete loss of functional *TP53* (bi-allelic deletion of *TP53* or mono-allelic deletion of 17p13 (del17p13) and *TP53* mutation) or 1q21 gain and International Staging System (ISS) stage 3 were defined as genomic high-risk (GHR) group; patients who had no markers of GHR group but were refractory or early relapse within 12 months were in FHR group; patients who did not fulfill any of the criteria for GHR or FHR were categorized into the SR group (13).

### NGS detection

The bone marrow (BM) aspirate samples were purified by positive selection of anti-CD138 magnetic microbeads (MiltenyiBiotec, Germany) in 141 NDMM patients. The other four patients without monoclonal plasma cells in BM used a biopsy sample of EMD. Genomic DNA was extracted from CD138+ cells using the QIAamp Blood DNA Mini Kit and biopsy samples using the GeneRead™ FFPE Kit (QIAGEN, Germany). AmpliSeq designed the MM panel for the Illumina Gene Assay with the Illumina Design Studio platform (https://designstudio.illumina.com/). It included 92 MM-related genes (**Supplementary File 1**), including *TP53* (16–18). Based on the manufacturer’s protocol, the library was constructed with an AmpliSeqTM Library PLUS for the Illumina kit (Illumina, USA). For each sample dataset, the mean sequence depth was above 1000x, and the 0.2x uniformity was not less than 0.85; otherwise, the library was reconstructed and sequenced. After library construction, sequencing was performed on an Illumina MiSeq Reagent Kit v3 (150 cycles) (Illumina, USA) DX system.

### Mutation analysis

For pre-processed data, the DNA Amplicon Analysis Module, installed on the Illumina Local Run Manager software and provided for free by Illumina, was used for transforming fastq files and calling single-nucleotide variation (SNV) and insertion/deletion (INDEL). Sequencing reads were aligned based on the human reference genome hg19 (GRCh37) using BWA v. 0.7.17 software. The indel realignment and base quality recalibration were performed to control quality using Gatk v.4.1.3.0. Local duplication alignment, base quality correction, and mutation analysis were performed on bam files using the analysis system’s Pisces software (Pisces 5.2.11.163). The ANNOVAR software performed the annotation for the Variant Call Format (VCF) (19). Databases of ExAC_ALL, gnomAD_ALL, and 1000G were used to identify single nucleotide polymorphisms (SNPs) that were excluded from further analysis. VAF filtered the gene mutations analyzed in this study≥5%, maximum in ExAC_ALL and gnomAD_ALL database less than 0.01%, record in COSMIC database hematology disease less than two and non-synonymous mutation (20).

### Statistical methods

The median values and ranges are reported for continuous variables, and proportions are reported for categorical variables. Variables with a normal distribution were analyzed with a two-sided t-test, while non-normal distribution variables were analyzed by Mann–Whitney U test. Categorical variables were analyzed using the Chi-square test and Fisher’s test. The survival analyses of PFS and OS were estimated and plotted by the Kaplan-Meier method. Fisher’s precision probability test examined the Gene Ontology (GO) cluster analysis and Gene Set Enrichment Analysis (GSEA) of gene mutation between two groups. All procedures were performed by R software v. 4.1.1 and GraphPad Prism v. 8.0, with a P value less than 0.05 regarded as significant.

## Results

### Baseline characteristics of FHR patients

Of 145 NDMM patients, there were 56 patients (38.6%) in the GHR group, 78 patients (53.8%) in the SR group, and 11 patients (7.6%) in the FHR group. The baseline clinical characteristics of patients in the three groups were summarized in **Table 1**. There were no differences between the three groups of age, ECOG score, or types of M protein. The GHR group had more patients with ISS III, R-ISS III, R2-ISS IV, and renal dysfunction, as expected. Moreover, the least partial anemia and hypercalcemia were observed in the SR group. We further analyzed these two groups separately to distinguish FHR from the SR group.

Interestingly, the FHR group was more likely to be male patients and coexisted with EMD and CPC. The induction regimens and ASCT received across the three groups were comparable. Nearly half of the patients had proteasome inhibitor (PI) and immunomodulatory (IMiD) combined regimens as first-line treatment, while the other half received PI-based regimens. The patients in SR groups acquired the best response compared with FHR and GHR groups (*P*<0.001).

### Shortest survival of FHR patients

**Figure 1:**
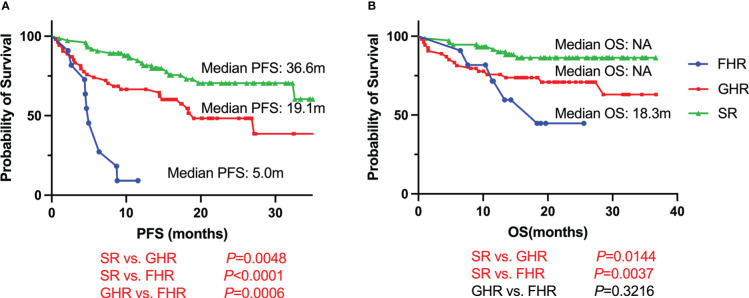
Survival curves for FHR, GHR, and SR NDMM patients. **(A)** PFS; **(B)** OS.

With a median follow-up time of 21.0 months (95% CI, 19.1-22.8 months), FHR groups had the shortest survival compared with GHR and SR groups (**Figure 1**). The median PFS was 5.0 months for FHR groups, while it was 19.1 months and 36.6 months for GHR and SR groups, respectively (*P*<0.0001). For the median of OS, the FHR group was similar to the GHR groups (18.3 months vs. not reach, *P*=0.3216), significantly poorer than the SR group (*P*=0.0037). The survival analysis of patients who received PI+IMiDs-based induction regimens showed similar results (**Supplementary Figure 3**). In the subgroup of only PI-based treatment, FHR patients had comparable PFS and OS compared with the GHR group, while significantly shorter than the SR group (**Supplementary Figure 3**).

### Treatment response and clinical outcomes of FHR patients

**Figure 2:**
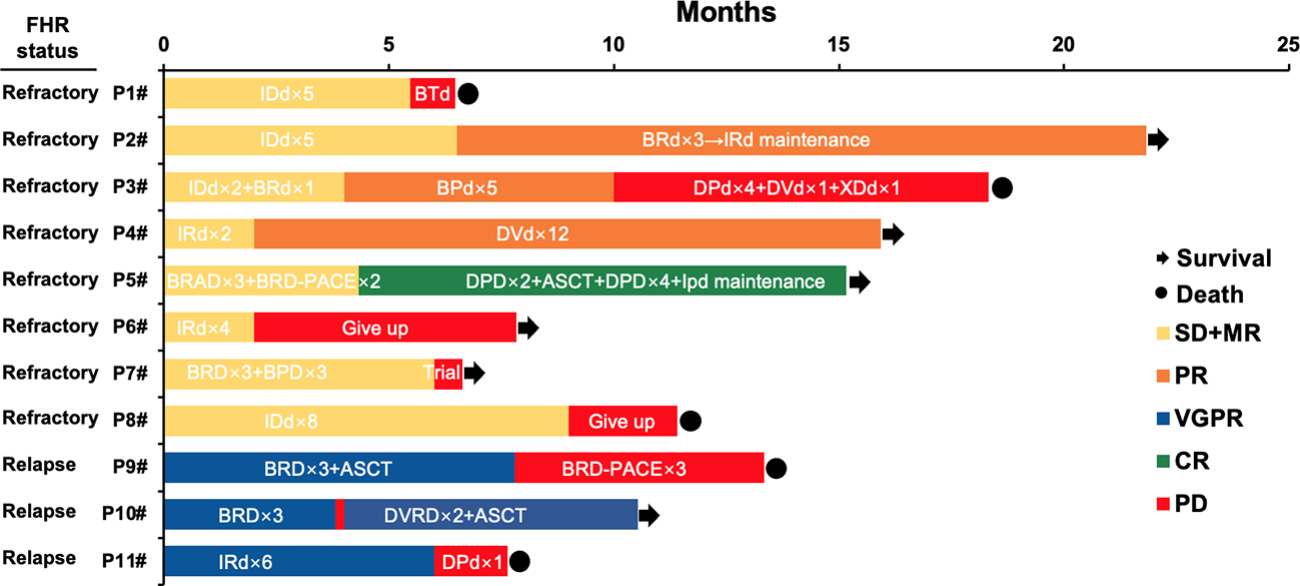
Treatment, response, and survival of eleven patients in the FHR group. IDd, Ixazomib- pegylated liposomal doxorubicin-dexamethasone; BTd, bortezomib- thalidomide-dexamethasone; BRD, bortezomib- lenalidomide-dexamethasone; BPD, bortezomib- pomalidomide-dexamethasone; DPd, daratumumab-pomalidomide-dexamethasone; DVD, daratumumab- bortezomib -dexamethasone; XDd, Selinexor (XPO1 inhibitor)- daratumumab-dexamethasone; IRD, Ixazomib- lenalidomide-dexamethasone; BRAD, bortezomib- lenalidomide- pegylated liposomal doxorubicin-dexamethasone; ASCT, autologous stem cell transplantation; IPD, Ixazomib- pomalidomide -dexamethasone; BRD-PACE, bortezomib- lenalidomide- dexamethasone- platinum- pegylated liposomal doxorubicin- cyclophosphamide- etoposide.

Eight refractory patients (8/11, 72.7%) and three patients relapsed in 12 months (3/11, 27.3%) in the FHR group. Among the eight refractory patients, 4 received PI, and IMiD combined first-line therapy, of which two changed to daratumumab-based second-line treatment and acquired CR and PR sustainably. The other two patients gave up and participated in a clinical trial respectively. The other four patients had PI-based regimens of induction therapy, of which one gave up and one adjusted to a daratumumab-based regimen. They sustained remission in PR, and two adjusted to PI and IMiD combined treatments and have died. For three early relapse FHR patients, all of them were treated with PI and IMiD combined induction therapy and acquired remission of VGPR. One of them added daratumumab in re-induction treatment and followed with ASCT resulting primary response of VGPR. Patient 9 was added chemotherapy drugs and died four months post PD. The other patient also adjusted to daratumumab-based second-line regimens and died from complications. See **Figure 2** for details.

**Figure 3:**
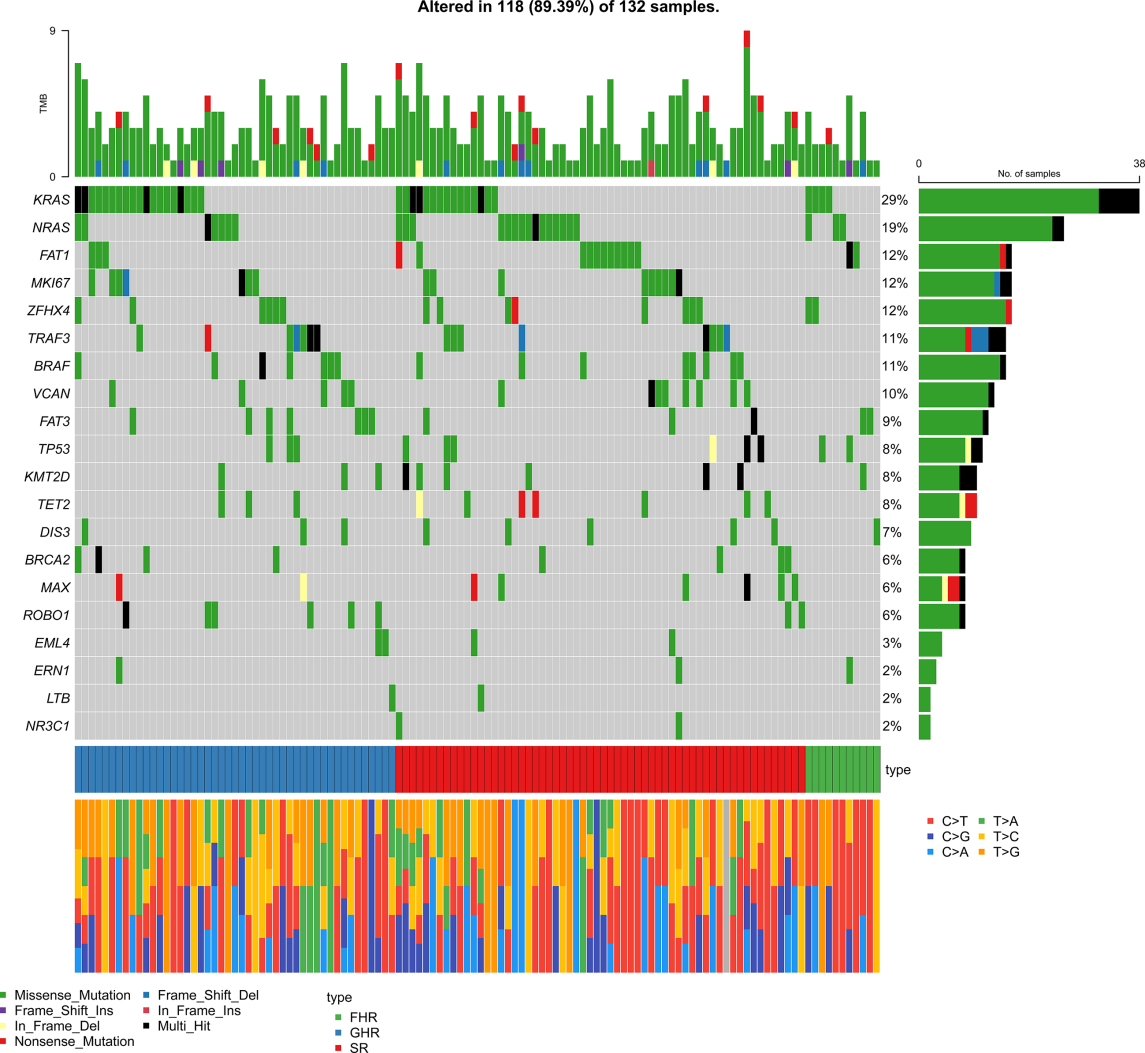
Composite heat map combining FISH detection and gene mutation.

### Genomic features of FHR patients

As the patients with amplification of 1q21 without ISS III or P53 mono-allelic inactivation were involved in the FHR group, we further analyzed the status of these two cytogenetic high-risk abnormalities (**Table 2**). The P53 mono-allelic inactivation of the FHR group was similar to that of the GHR group, significantly higher than that of the SR groups (*P*=0.038). There was no apparent differentiation of amplification of 1q21 between FHR and the other two groups. The gene mutation analysis showed that the FHR group had higher mutation frequencies of *MKI67* than the SR and GHR groups (45% vs. 15% vs. 9%). Meanwhile, *ERN1*(18% vs. 0, *P*=0.014) and *EML4* (18% vs. 1%, *P*=0.039) were predominantly mutated in FHR than in the SR group. The number of mutations was similar in the three groups.

**Table 2:** Subgroup analysis of genetic abnormalities.

	Number (Percentage, %)	*P* value
FHR vs. SR		
Amp 1q21	5/11 (45) vs. 19/78 (24)	0.158
P53 mono-allelic inactivation	3/11 (27) vs. 4/78 (5)	**0.038**
*MKI67* mutation	5/11 (45) vs. 12/78 (15)	**0.032**
*ERN1* mutation	2/11 (18) vs. 0/78 (0)	**0.014**
*EML4* mutation	2/11 (18) vs. 1/78 (1)	**0.039**
FHR vs. GHR		
Amp 1q21	5/11 (45) vs. 38/56 (68)	0.182
P53 mono-allelic inactivation	3/11 (27) vs. 13/56 (23)	0.545
*MKI67* mutation	5/11 (45) vs. 5/56 (9)	**0.008**
SR vs. GHR		
Amp 1q21	19/78 (24) vs. 38/56 (68)	**<0.001**
P53 mono-allelic inactivation	4/78 (5) vs. 13/56 (23)	**0.003**
*TP53* mutation	0/78 (0) vs. 9/56 (16)	**<0.001**
*CDKN1B* mutation	0/78 (0) vs. 4/56 (7)	**0.029**

FHR, functional high-risk; SR, standard risk; GHR, genetically high-risk. The bold values was presented for P > 0.05.

**Figure 4:**
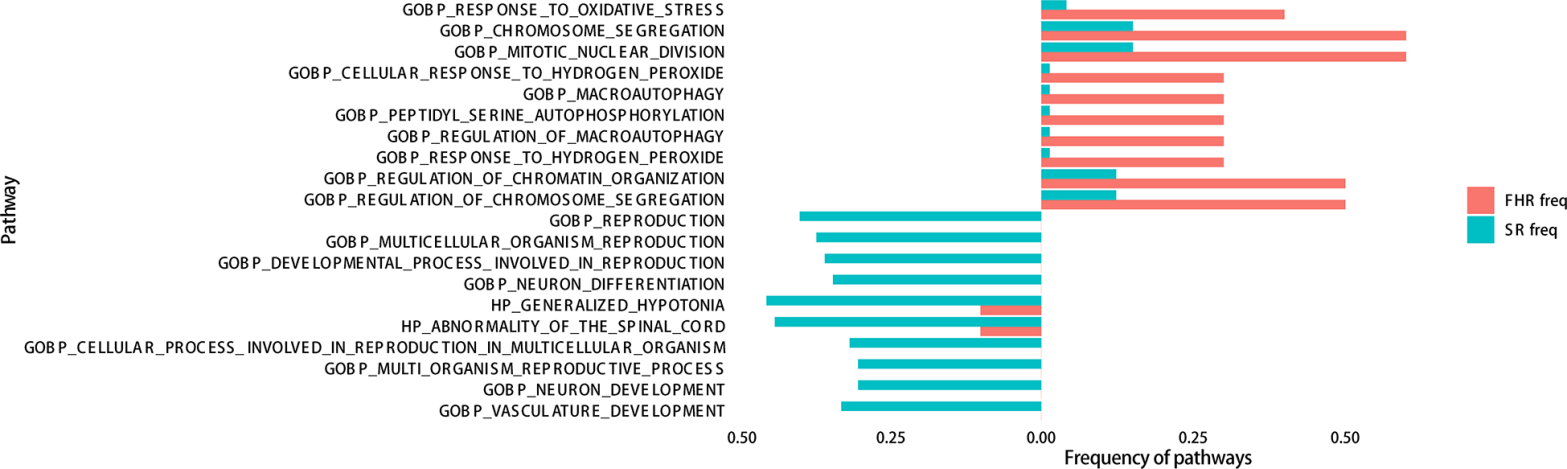
GO enrichment of the top 20 genes in FHR and SR groups analyzed by Fisher’s test.

To distinguish FHR patients from the SR group, we analyzed the enrichment of gene mutations between the FHR and SR groups. The differential mutation genes were markedly enriched in “chromosome segregation, miRNA regulation” of the biological process (BP) category, “spindle, mitochondrial matrix” of the cellular component (CC) category, and “DNA binding transcription” of the molecular function (MF) category (**Supplementary Figure 1**). Considering fewer differential genes calculated in GO analysis, we involved all mutated genes and enriched for gene sets of FHR and SR groups, then analyzed with Fisher’s exact test for differences. The top ten pathways of each group that were significantly enriched are listed in **Figure 4**. The “response to oxidative stress” (*P*=0.0033) of BP was most improved in the FHR group, while “reproduction” (*P*=0.0126) was in the SR group. The GSEA analysis showed that the PI3K-Akt pathway seems to enrich in the FHR group with a higher gene ratio (**Supplementary Figure 2**), which had no statistical difference from the SR group.

## Discussion

This real-world retrospective study on FHR NDMM patients showed that the FHR group with the shortest survival had specific clinical characteristics of males coexisting with EMD and CPC. As FHR patients do not harbor high-risk cytogenetic abnormalities defined by R-ISS and double-hit, they are always confused with the SR group and manifest after induction therapy until 12 months follow-up. Identifying FHR patients at diagnosis might help to choose an appropriate therapeutic schedule to improve clinical outcomes. Therefore, we further explored the gene mutation signatures of FHR patients and found increased mutations in several genes affecting chromosome segregation et al. Meanwhile, whether patients with P53 mono-allelic inactivation should be involved in the FHR group remains to be verified.

The definition of FHR was promoted by Spencer in 2019, which included sub-optimal responders (SOR) of MR or SD and early progressors (EP) within 12 months of commencing first-line therapy, irrespective of cytogenetic status (12). With a median follow-up of 23 months among 1320 NDMM patients, Spencer reported 11.5% was SOR, and 8.9% was EP, in which 25% was also demonstrated SOR. On that basis, Chng et al. analyzed the CoMMpass dataset of 406 NDMM patients and further clarified FHR patients without GHR, in which 15% were FHR, 26.1% were GHR, and 58.9% were SR (13). Compared to these two database studies, we found that there were 7.6%, 38.6% and 53.8% in FHR, GHR and SR groups in real-life data. The lower proportion of the FHR group probably is because of the deletion of non-relapse deaths in 12 months and progression due to withdrawal, which is hard to recognize in the database. Therefore, our research found more SOR (8/145, 5.5%) than EP (3/145, 2.1%), indicating that early recognition of FHR patients and an appropriate first-line regimen might improve clinical outcomes.

The clinical characteristics might be helpful to distinguish FHR patients at diagnosis. A previous study on early relapse reported that patients with ISS III, refractory to novel triplet agents of induction therapy and no maintenance treatment post-ASCT were at high risk of relapse in 24 months post-ASCT (21). The other researchers analyzed the trail of Myeloma XI and found NDMM patients relapsed in 12 months post-ASCT more likely with anemia, hypercalcemia, ISS III, gain 1q, t(4;14) and del 17p (22). Despite tumor burden and high-risk cytogenetic abnormalities, case paired data from China revealed that early relapsed within 12 months was characterized by with an increased expression of CPCs (23). A further study reported two baseline factors associated with SOR of age >70 years old and induction regimen without PIs (12). In contrast, indicators of the higher disease burden are associated with EP (12). Restricted to fewer clinical data from the database, Chng et al. failed to find unique clinical characteristics of FHR patients except for genetic features (13). In this study, we demonstrated that FHR patients were more likely to be male and had EMD and CPC at diagnosis, which had been well-established as high-risk clinical features (6). This finding might help identify FHR patients from standard cytogenetic groups and benefit from risk-adapted therapy.

The first line treatment of our cohort was half PI+IMiD-based and half PI based, comparable to the population from the compass database (13). The median OS of FHR groups analyzed from the database was 27.6 months, which is better than what we reported of 18.3 months. The high proportion of refractory patients and no withdrawal-related relapse in our FHR groups might lead to the worst survival compared with previous studies. The patients in the FHR group acquired the worst response of the three groups, under the comparable treatment regimen. The subgroup survival analysis showed that PI+IMiDs might improve the PFS of GHR patients, rather than FHR patients. The further analysis of each FHR case one by one indicated that a daratumumab-based induction regimen might improve the treatment response and survival. Otherwise, the first-line regimen recommended by NCCN of carfilzomib, lenalidomide, and dexamethasone might overcome the refractory of FHR patients (24). The available treatment options are almost entirely based on high-risk cytogenetic signatures (15, 25), so new agents and more clinical trials explicitly targeting FHR groups are needed to explore a more specific therapeutic strategy.

Despite clinical characteristics, genomics and biology features may provide insights into distinguishing points and potential therapeutic targets for FHR patients. Chng et al. analyzed transcriptome data and copy number aberrations (CNAs), resulting in no high-risk gene expression signature reported previously; further proposed factors outside of the tumor cells, including the tumor microenvironment, may play an essential role in FHR (13). The mutation status analysis indicated that FHR patients had increased mutations affecting the *IL-6/JAK/STAT3* signaling pathway with predominantly mutated *KIAA1549L*, *LUZP2*, and *BMPR1B* (13). In our trial, patients received target gene sequencing which acquired fewer data than the whole-exome sequencing they used, which accompanied a severe class imbalance. We found gene mutations of *EML4*, *MKI67*, and *ERN1*, primarily in FHR patients. The GO analysis also pointed to oxidative stress, chromosome segregation, and hypoxia tolerance, which were revealed as new MM molecular resistance pathways by single-cell sequencing (26). Further analysis of GSEA proposed gene mutation enriched in the *PI3K/AKT* pathway, while deficient in FHR cases resulting in no statistical difference compared with SR patients. The *PI3K/AKT* signaling has been identified as necessary for MM cell survival and growth, affecting stromal cells (27). However, more focused studies addressing genetic signatures remain necessary to elucidate signaling pathways and potent inhibitors for FHR patients.

Back to the definition of the FHR group, Chng et al. excluded amplification of 1q21 without ISS III and P53 mono-allelic inactivation, which are generally considered high-risk factors by R-ISS, IMWG, and SMART risk stratification (2, 28, 29). In our real-world cohort, FHR patients had more P53 mono-allelic inactivation than SR patients and comparable levels to the GHR group, indicating patients with P53 mono-allelic inactivation might belong to GHR. Otherwise, FHR also coexisted with the high-risk clinical features of EMD and CPC, which could be generally considered high-risk patients at diagnosis. The definition of FHR may need to be revised and more data collected to validate it.

In conclusion, FHR NDMM patients exist in the real world. They presented the shortest survival, even without any high-risk genetic abnormalities in the era of PI and IMiDs induction regimen. FHR patients were characterized by the clinical features of males, accompanied by EMD and CPC, and also had increased mutations affecting the biological processes of oxidative stress, chromosome segregation, and hypoxia tolerance. These clinical and genetic signatures may help to recognize FHR patients at diagnosis and explore new treatment strategies for these patients.

## Data availability statement

The raw data supporting the conclusions of this article will be made available by the authors, without undue reservation.

## Ethics statement

The studies involving human participants were reviewed and approved by the ethics committee of Beijing Jishuitan hospital. The patients/participants provided their written informed consent to participate in this study. Written informed consent was obtained from the individual(s) for the publication of any potentially identifiable images or data included in this article.

## Author contributions

Conception and design: LB, Y-TW. Provision of study materials and patients: BC, M-QL, LS, SG, L-JF, Q-QX. Collection and assembly of data: Y-TW, M-ZW. Data analysis and interpretation: Y-TW, T-GZ, KS. Manuscript writing: Y-TW, LB. Final approval of manuscript: All authors. Accountable for all aspects of the work: All authors.

## Supplementary material

Supplementary Figure 1 Bubble diagram of GO enrichment in gene mutations of FHR group.

Supplementary Figure 2 Bubble diagram of GSEA enrichment in gene mutations of FHR group.

Supplementary Figure 3 Survival curves for subgroups of FHR, GHR, and SR NDMM patients with different induction regimens. PFS (A) and OS (B) for patients who received PIs+IMiDs-based regimens; PFS (C) and OS (D) for patients who received PIs-based regimens.
