# Supplementary figures and images for: Clinical and genomic characterization of Chinese patients with functional high-risk multiple myeloma: A real-world validation study
Source: Front Oncol. 2023 Mar 10;13:1110693. doi: 10.3389/fonc.2023.1110693 (PMC10036342; doi:10.3389/fonc.2023.1110693)

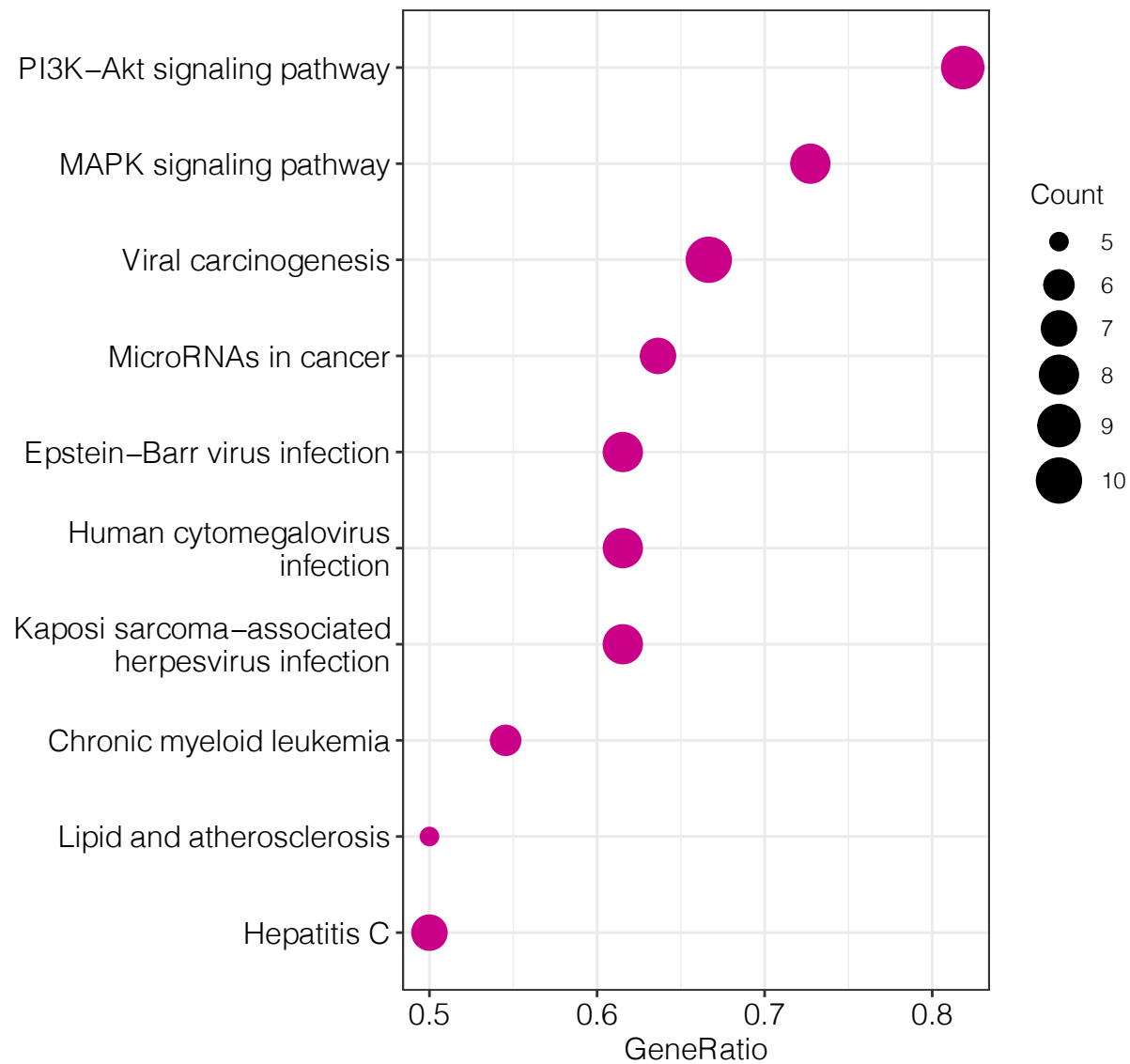

Supplement: Supplementary Figure 1 — Bubble diagram of GO enrichment in gene mutations of FHR group. [file DataSheet_1.pdf]

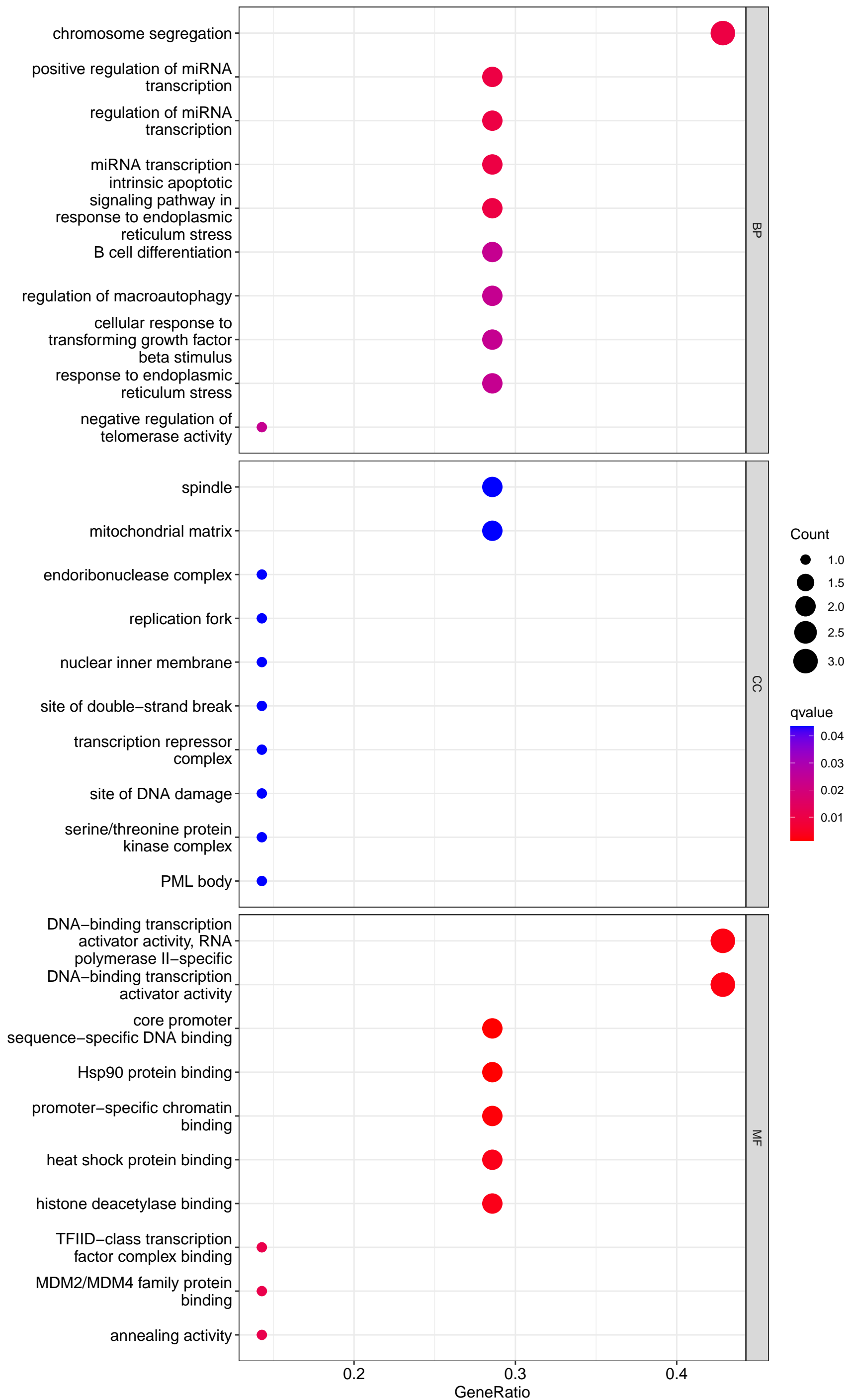

Supplement: Supplementary Figure 2 — Bubble diagram of GSEA enrichment in gene mutations of FHR group. [file DataSheet_2.pdf]

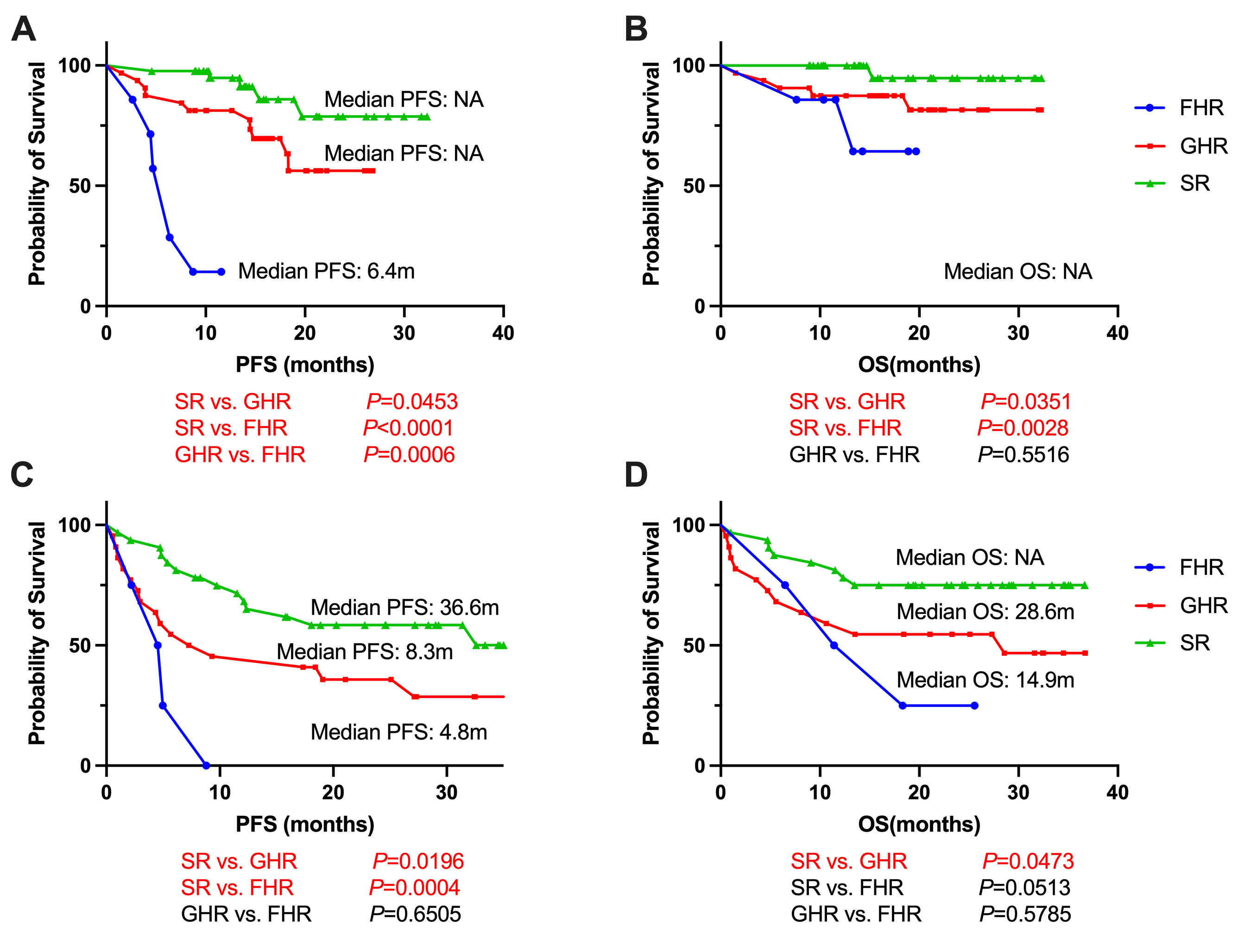

Supplement: Supplementary Figure 3 — Survival curves for subgroups of FHR, GHR, and SR NDMM patients with different induction regimens. PFS (A) and OS (B) for patients who received PIs+IMiDs-based regimens; PFS (C) and OS (D) for patients who received PIs-based regimens. [file Image_1.tiff]
