# Supplementary material for: Clinical and genomic characterization of Chinese patients with functional high-risk multiple myeloma: A real-world validation study
Source: Front Oncol. 2023 Mar 10;13:1110693. doi: 10.3389/fonc.2023.1110693 (PMC10036342; doi:10.3389/fonc.2023.1110693)
Supplement: Supplementary file 4 [file Table_1.docx]

Supple Table 1. 92 Gene detection of NGS panel

| ACTG1 | AKT1 | ALK | ATM | BIRC2 | BIRC3 | BRAF |
| --- | --- | --- | --- | --- | --- | --- |
| BRCA2 | CARD11 | CCND1 | CCND3 | CDK4 | CDKN1B | CDKN2A |
| CDKN2C | CRBN | CSF3R | CUL4A | CUL4B | CUX1 | CXCR4 |
| CYLD | DDB1 | DIS3 | DNMT3A | EGFR | EGR1 | EML4 |
| EP300 | ERN1 | FAM46C | FAT1 | FAT3 | FAT4 | FBXW7 |
| FGFR3 | FOXO1 | GFI1 | GNAQ | HIST1H1E | IKZF1 | IKZF3 |
| IRF4 | JAK1 | JAK2 | KDM6A | KIT | KLHL6 | KMT2D |
| KRAS | LRP1B | LTB | MAF | MAFB | MAX | MDM2 |
| MKI67 | MUM1 | MYC | MYD88 | NFKB2 | NR3C1 | NRAS |
| PCDH10 | PIK3CA | PRDM1 | PRKD2 | PSMB5 | PSMB9 | PSMG2 |
| PTEN | PTPN1 | RASA2 | RB1 | RIPK1 | ROBO1 | ROBO2 |
| RUNX1 | RUNX3 | SOCS3 | SP140 | STAT3 | SUZ12 | TET2 |
| TNFRSF21 | TP53 | TRAF2 | TRAF3 | VCAN | WHSC1 | XBP1 |
| ZFHX4 |  |  |  |  |  |  |

In 145 samples of newly diagnosed multiple myeloma patients, 132 samples (91%) detected at least one gene mutation. Of 92 genes we designed in this target gene NGS panel, 74 genes (80%) were detected at least in one patient.
